# Effects of dietary nutmeg (*Myristica fragrans*) seed meals on growth, non-specific immune indices, antioxidant status, gene expression analysis, and cold stress tolerance in zebrafish (*Danio rerio*)

**DOI:** 10.3389/fnut.2022.1038748

**Published:** 2023-01-26

**Authors:** Farzaneh Vakili, Zahra Roosta, Roghieh Safari, Mojtaba Raeisi, Md. Sakhawat Hossain, Inês Guerreiro, Arash Akbarzadeh, Seyed Hossein Hoseinifar

**Affiliations:** ^1^Department of Fisheries, Sari Agricultural Sciences and Natural Resources University, Sari, Iran; ^2^Fisheries Department, Faculty of Natural Resources, University of Guilan, Someh Sara, Gilan, Iran; ^3^Department of Fisheries, Faculty of Fisheries and Environmental Sciences, Gorgan University of Agricultural Sciences and Natural Resources, Gorgan, Iran; ^4^Food, Drug and Natural Products Health Research Center, Golestan University of Medical Sciences, Gorgan, Iran; ^5^Hagerman Fish Culture Experiment Station, University of Idaho, Hagerman, ID, United States; ^6^CIIMAR - Interdisciplinary Centre of Marine and Environmental Research, Terminal de Cruzeiros do Porto de Leixões, University of Porto, Matosinhos, Portugal; ^7^Department of Fisheries, Faculty of Marine Science and Technology, University of Hormozgan, Bandarabbas, Iran

**Keywords:** nutmeg, growth and immune related genes, oxidative stress, coldwater stress challenge, medicinal herb

## Abstract

**Introduction:**

A medicinal plant, *Myristica fragrans* seed meal (nutmeg), was utilized to evaluate its impact on the growth, immunity, and antioxidant defense of zebrafish (*Danio rerio*).

**Methods:**

In this regard, zebrafish (0.47 ± 0.04 g) (mean ± S.D.) were fed with 0% (control), 1% (T1-nutmeg), 2% (T2-nutmeg), and 3% (T3-nutmeg) of powdered nutmeg for 70 days. At the end of the feeding trial, growth performance, survival rate of fish, and temperature-challenge effects were recorded. Immune and antioxidant parameters were also assessed through the collection of serum and skin mucus samples.

**Results:**

The results indicated that nutmeg supplementation did not significantly influence the growth of zebrafish (*P* > 0.05); however, the survival rate of fish fed with 2 and 3% of nutmeg supplementation significantly decreased (*P* < 0.05). The skin mucus and serum total protein, total immunoglobulin (Ig), and lysozyme activity were significantly increased in T3-nutmeg treatment in comparison to the control (*P* < 0.05). Superoxide dismutase (SOD) and catalase (CAT) activities were also enhanced in the T3-nutmeg group (*P* < 0.05). Nutmeg supplementation significantly upregulated the mRNA expression of *growth hormone (gh)* and *insulin growth factor-1* (*igf-1*). Moreover, the nutmeg inclusion upregulated the expression of *interleukin-1*β (*IL-1*β), *lysozyme, sod*, and *cat*. The dietary supplementation of nutmeg significantly increased the resistance of zebrafish against cold-water shock and survivability afterward (*P* < 0.05).

**Discussion:**

In conclusion, the supplementation of 3% powdered *nutmeg* in zebrafish diets could be suggested as an effective immune stimulator that improves antioxidant defense and stress tolerance.

## 1. Introduction

It is crucial to enhance fish welfare, especially in intensive aquaculture, to achieve high production to meet market requirements (1). In high-density fish farms, however, various instabilities are likely to occur, for example, fluctuations in water quality, which can lead to fungal and bacterial outbreaks (2, 3). Immunostimulants in aqua-feeds are becoming an increasingly promising technique for coping with stressors and disease outbreaks in fish farms, and among them, there are several plant-based additives that can potentially improve body performance in the aquaculture industry (4–7). The therapeutic roles of medicinal plants, such as anti-inflammatory, antimicrobial, antioxidant, and anti-cancer, make them popular remedies (8). Natural products have thousands of bioactive components (phenolic agents and flavonoids) derived from different parts well-known as immune boosters (9–11). The non-specific immune system, *i.e.*, epidermis and their agents, besides cellular and humoral factors, for example, chemical mediators (cytokines), lysozyme, lectins, complement, and protease, are shown to be activated by plants (12–14). Extracted, oiled, and crude powdered herbal medicines could potentially be incorporated into fish’s diets (7, 15, 16), which are considered an alternative approach for antibiotics with no adverse effects on either animals and/or the ecosystem (15, 17).

The nutmeg is the seed of *Myristica fragrans*, which is well-known as a spice and functions as a natural antioxidant by preventing the peroxidation of lipids and quenching singlet oxygen (18). A variety of chemical components and active phytochemicals, including vitamins, alkaloids, phenolic, flavonoids, and lignan, improve their antioxidant effects against oxidative stress (19). *M. fragrans* is a plant with antithrombotic, antifungal, antibacterial, and anti-inflammatory properties (20, 21) that were reported to be effective when mixed with other plants, for example, garlic, ginger, and rosemary (22, 23). In fish, some adverse effects of dietary nutmeg extract are reported in both the growth and blood biochemistry of rainbow trout (*Oncorhynchus mykiss*) (24), and nutmeg-supplemented diets have been shown to increase the growth and antioxidant parameters in common carp (*Cyprinus carpio*) (25). To the best of our knowledge, the effectiveness of nutmeg on the regulation of genes has not been investigated in any fish species. Therefore, the present study examined the effects of nutmeg powder at different doses on growth performance, serum and skin mucus non-specific immunity, antioxidant capacity, and expression of genes related to immunity, growth, and antioxidant, in addition to stress tolerance in zebrafish (*Danio rerio*).

## 2. Materials and methods

### 2.1. Experimental diets and growth trial

The powder form of nutmeg (*M. fragrans*) was obtained from the Food, Drug, and Natural Products Health Research Center (Golestan University of Medical Sciences, Gorgan, Iran), and then added to the experimental prepared diets. The ingredients and proximate composition of the control diet are presented in Table 1. For 70 days, powdered nutmeg was added to the control diet in 1% (T1-nutmeg), 2% (T2-nutmeg), and 3% (T3-nutmeg) doses. The ingredients were mixed, extruded, and dry pelleted, and then stored at 4°C during the experimental trial.

**TABLE 1:** Basal diet ingredient composition and proximate composition (%).

Ingredients	% Dry weight basis	Proximate composition (% dry matter basis)	
Fish meal	40.0	Dry matter	92.22
Wheat flour	21.0	Crude protein	36.70
Soybean meal	13.5	Crude lipid	11.13
Gluten	5.5	Ash	3.48
Soybean oil	6.0		
Fish oil	6.0		
Mineral premix^a^	3.0		
Vitamin premix^a^	2.0		
Binder^b^	2.0		
Anti fungi^c^	0.5		
Antioxidant^d^	0.5		

^a^Permix detailed by 58; ^b^Amet binder™, Mehr Taban, Yazd, Iran; ^c^ToxiBan antifungal (Vet-A-Mix, Shenan-doah, IA). ^d^Butylated hydroxytoluene (BHT) (Merck, Germany).

In order to determine the chemical composition of the essential oil of nutmeg (Table 2), a Clevenger-type apparatus was used to hydro-distill the essential oil (EO) and is stored in the dark at 4°C for further analysis. Gas chromatography (GC) was used to identify the chemical composition of MF EOs based on the following parameters: 1.5 mL/min of helium flow rate and temperature increased from 40 to 240°C with a gradient of 3°C/min. Following the initial and final temperature holding periods of 6 min, the temperature was raised to 300°C at 15°C/min.

**TABLE 2:** Chemical composition of essential oil of *Myristica fragrans* using GC/MS analysis.

Number	Component	Retention time (min)	Total (%)
1	α Thujene	3.24	1.24
2	α Pinene	3.43	3.80
3	Camphene	3.92	1.20
4	Sabinene	4.12	38.14
4	Beta pinene	4.64	7.11
5	α Phellandrene	4.81	3.21
6	Cymene	5.13	3.14
7	Limonene	5.24	6.49
8	Beta ocimene	5.43	0.28
9	Gamma terpinene	5.70	6.80
10	α terpinolene	6.02	2.03
11	Linalool	6.13	1.15
12	Terpeniol	6.42	0.70
13	Mentha tiene	6.89	0.17
14	α terpineol	7.17	0.10
15	Borneol acetate	7.43	0.19
16	Isosafrole	7.79	2.80
17	Terpinene acetate	8.25	0.50
18	Anisole	8.73	1.31
19	Eugenol	9.14	0.62
20	Copaene	9.93	2.13
21	Cyclohexene	10.28	0.25
22	Caryophyllen	10.72	0.70
23	Isoeugenol	11.62	0.20
24	Germacrene	12.14	0.73
25	Cis methyl isoeugenol	12.80	1.06
26	Myristicine	13.30	9.30
27	Alpha humulene	13.41	0.10
28	Elemicin	13.72	1.80
Total number			97.25%

A total of 600 zebrafish (*D. rerio*) fries (0.47 ± 0.04 g) (mean ± S.D.) were obtained from Gorgan Mahi hatcheries. Before the feeding trial, fish were randomly distributed into 12 glass (100 × 40 × 30 cm) aquaria (50 fish per aquarium; 100 L) fed with a commercial diet (BioMar^®^-France) and acclimated for around 1 week. Zebrafish were fed until apparent visual satiation thrice daily at 8:00 A.M., 2:00 P.M., and 8:00 P.M. during 70 days of feeding trial. Based on the Gorgan Mahi hatcheries’ system, temperature (23 degrees Celsius), pH (7.5), and dissolved oxygen (7.5 mg L^–1^) were regularly monitored using Eutech Instrument, PH 450 digital.

### 2.2. Sampling and growth parameters

According to “Zebrafish Lab” instructions from the Gorgan University of Agricultural Sciences and Natural Resources, the sampling procedures of this project were carried out in compliance with the Instruction of the Ethical Committee for Animal Care and Use.

At the end of the feeding trial, fish were sampled for body performance analysis. The weight of the fish was measured to the nearest 0.01. Growth performance and survival rate were measured in each treatment using the following formulas:

Weight gain (WG, g) = final weight – initial weight

Survival rate (SR, %) = 100 × [final number of fish/initial number of fish].

After 70 days of the feeding trial, nine fish per treatment were individually transferred to a polyethylene bag containing 10 ml of 50 mM NaCl (Sigma, Steinheim, Germany) and gently rubbed for approximately 30 s, then pooled mucus samples from each container were collected in 10-ml tubes (centrifuged at 1500 × *g*, 10 min, 4°C) and stored at −80°C until enzymes analysis. Fish were then anesthetized using clove powder (0.5 g L^–1^), the head and caudal fin were removed, and fish were placed immediately in liquid nitrogen, and then stored at −80°C. Samples were homogenized by PBS, then centrifuged (at 3000 × *g*, 4°C) to collect the supernatant. The extracted whole-body serum was performed according to (26, 27) with some modifications, and then was kept at −18°C until further analysis.

### 2.3. Immune and antioxidant parameters

Total protein levels were measured by adding (5–10) μl of mucus and serum samples to the diluted reagents. After a 5-min interval, protein content was detected and read over 60 min at 546 nm, based on the instruction of Pars Azmoun Commercial kit (Karaj, Iran).

Total immunoglobulin (Ig) levels of mucus and serum were measured following (28) with slight modification. First, the total protein levels were measured as described earlier. Then, Ig molecules were precipitated by a 12% polyethylene glycol solution (Sigma), and the protein content of the samples was measured again. The difference in protein contents before and after precipitation with polyethylene glycol was considered the total Ig content of skin mucus and serum.

The lysozyme activity in skin mucus and serum was measured as described by Subramanian et al. (29). *Micrococcus luteus* (ATCC 4698) bacteria were used as the enzyme assay substrate. Absorption of the solution was measured over negative and positive controls. A unit of enzyme activity is equal to the enzyme at 450 nm and decreased by 0.001 per min.

The activities of antioxidant enzymes, superoxide dismutase (SOD), and catalase (CAT) were measured using commercially available kits (ZellBio GmbH, Hinter den Garten 56, Lonsee, Germany).

### 2.4. Growth-, immune-, and antioxidant-related gene expression

#### 2.4.1. RNA extraction and cDNA synthesis

After the end of the feeding trial, brain, liver, and intestine tissues from nine specimens of each treatment (described in the section “2.2. Sampling and growth parameters”) were dissected, deep-frozen in liquid nitrogen, and then stored at −80°C for further RNA isolation. Total RNA was isolated from 50 to 100 mg of tissue using an Esterabad-Zistfan-Pishro-Azma RNA extraction kit. According to the manufacturer’s instructions, total RNA was treated with DNase I (Fermentas, Lithuania) to avoid contamination with genomic DNA. Then, the quantity and quality of RNA were then assessed using NanoDrop (Nanodrop technology, Wilmington, DE, U.S.A.) at 260/280 nm and loading RNA on 1.5% denatured agarose gel to evaluate the 28S and 18S rRNAs, observing no smear and two intensive bands at approximately 750 and 1200 bp with the ratio of intensities about 2:1 indicating successful RNA preparation. Afterward, the extracted RNA was reverse-transcribed into cDNA using a cDNA synthesis kit (Fermentas, Lithuania). To detect the possibility of genomic DNA contamination, intron spanning primer (B-actin with intron) was used.

#### 2.4.2. Primers and real-time PCR

The sequences of primers used in real-time PCR (Table 3) were selected from our previous studies (30). Quantitative real-time PCR (qPCR) was performed using an iCycler (Bio-Rad, U.S.A.) with Fermentas Maxima SYBR Green qPCR Master Mix (Fermentas) and gene-specific primers following thermal profile: 94°C during 5 min, 40 cycles at 10 s at 94°C, followed by 10 s at 59°C and 10 s at 72°C and each reaction was amplifying in triplicate (Table 2). The β-actin stability has been validated as a reference gene based on previous studies in zebrafish organs, to normalize the expression of the target genes (31). The fold change in relative *growth hormone (gh)*, *insulin growth factor-1* (*igf-1*), *interleukin-1*β (*IL-1*β), *lysozyme*, *sod*, and *cat* expression was calculated by the 2^–ΔΔCt^ method, where delta Ct = (delta Ct of treated - delta Ct of control), and a standard curve is also used to assess the performance of qPCR assay by estimating its efficiency (32). All the data were analyzed by the Bio-Rad System software, version 2.00 (Hercules, CA, USA).

**TABLE 3:** Primers sequences (F, forward; R, reverse) used for real-time PCR of zebrafish (*Danio rerio*).

Primers name	Primers sequences	accession no.	Application	Amplified fragment length	TM (°C)	Efficiency (%)
*gh* *gh*	F:TTGGTGGTGGTTAGTTTGCT R:CTCAACTGTCTGCGTTCCTC	AJ937858.1	Brain	160	59	95%
*Igf-1* *Igf-1*	F:AGTGTACCATGCGCTGTCTC R:AATAAAAGCCCCTGTCTCCA	NM_131825.2	Liver	158	59	95%
*cat* *cat*	F:GCATGTTGGAAAGACGACAC R:GTGGATGAAAGACGGAGACA	AJ007505.1	Liver	195	59	95%
*sod* *sod*	F:GGGTGGCAATGAGGAAAG R:GCCCACATAGAAATGCACAG	BC055516.1	Liver	250	59	95%
*IL-1*β *IL-1*β	F:CGTCTCCACATCTCGTACTCA R:GTGTCTTTCCTGTCCATCTCC	AY340959.1	Intestine	197	59	95%
*lysozyme* *lysozyme*	F:GGCAGTGGTGTTTTTGTGTC R:CGTAGTCCTTCCCCGTATCA	NM_139180.1	Intestine	202	59	95%
β*-actin* β*-actin*	F:AGCAGATGTGGATCAGCAAG R:TACCTCCCTTTGCCAGTTTC	NM_131031.1	All samples	205	59	95%
β*-actin with intron* β*-actin with intron*	F:TCCCCACAACTTCCAGAG R:TACCTCCCTTTGCCAGTTTC	ENSDARG00000037746	Determining of DNA contamination	550	59	

Primer names: *gh*, growth hormone; *igf*-1, insulin-growth-factor-1; *cat*, catalase; *sod*, superoxide dismutase; *IL-1*β, interleukin-1β; *lysozyme*.

### 2.5. Coldwater stress challenge

At the end of the feeding trial, 15 zebrafish from each tank were randomly selected and checked for health status by monitoring their swim behavior. The fish were then kept in four-liter containers to determine the mortality rate (M.R.%) when cold-shocking was applied to the trial specimens. Water temperature was manipulated down to around 10 ± 2°C below the temperature of the original tank temperature following the method described by (33). M.R. of each treatment in triplicate (13 ± 2°C) was recorded after 4 h.

### 2.6. Statistical analysis

After checking the normality of data (Kolmogorov–Smirnov test) and homogeneity of variance (Levene’s test), one-way analysis of variance (ANOVA) with α = 0.05 was used for data analysis. Data are represented as mean ± standard error (S.E). Independent-samples *t*-test was used to compare parameters between the mucosa and body serum levels for each feeding treatment. The SPSS statistical package, version 25.0 (SPSS Inc., I.B.M. Co., Armonk, NY, USA) was used.

## 3. Results

### 3.1. Growth performance and survival

Table 4 presents the results of weight gain (WG) and survival rate (SR) of zebrafish at the end of a 70-day trial. There were no significant differences in growth performance among zebrafish fed the nutmeg supplementary diets with different doses of plant and the control group (*P* > 0.05). Zebrafish fed with 2 and 3% supplementation of nutmeg revealed a significantly lower survival rate than the control group (*P* < 0.05).

**TABLE 4:** Growth performance and survival rate of zebrafish fed the experimental diets.

Body performance	Diets			
	Control	T_1–nutmeg_	T_2–nutmeg_	T_3–nutmeg_
Final weight (g)	0.80 ± 0.05^a^	0.83 ± 0.03^a^	0.80 ± 0.05^a^	0.83 ± 0.03^a^
Weight gain (g)	0.33 ± 0.08	0.36 ± 0.05	0.33 ± 0.06	0.36 ± 0.06
Survival rate (%)	84.66 ± 2.90^a^	79.33 ± 1.76^ab^	75.33 ± 2.90^b^	71.33 ± 2.40^b^

Data presented as the mean ± S.E. Different small letters denote significant differences among treatments (*P* < 0.05).

### 3.2. Immune and antioxidant responses

Table 5 presents the effects of dietary nutmeg on zebrafish skin mucus and serum levels after 70 days of feeding. Total protein levels of both mucus and serum were significantly increased in fish fed with 3% nutmeg supplementation (*P* < 0.05). Similarly, total Ig levels of mucus were significantly higher in fish fed with T3-nutmeg compared to the control group (*P* < 0.05). The total Ig level of serum was significantly higher in the fish T2-nutmeg-treated group than in the control (*P* < 0.05). The mucosal lysozyme activity was significantly higher in fish fed with 3% nutmeg in comparison to the control group, while the body serum lysozyme was not significantly different between the zebrafish fed with nutmeg and the control group (*P* > 0.05).

**TABLE 5:** Immune indices [total protein, total immunoglobulin (Ig), and lysozyme activity] and antioxidant activity [superoxide dismutase (Sod) and catalase (Cat)] of zebrafish fed the experimental diets.

Variables	Samples	Diets			
		Control	T_1–nutmeg_	T_2–nutmeg_	T_3–nutmeg_
Total protein (mg ml^–1^)	Serum	*1.76 ± 0.14^b^	*1.76 ± 0.33^b^	*2.00 ± 0.05^ab^	*2.36 ± 0.08^a^
Total protein (mg ml^–1^)	Mucus	0.78 ± 0.44^b^	0.95 ± 0.02^ab^	1.03 ± 0.08^ab^	1.15 ± 0.08^a^
Total Ig (mg ml^–1^)	Serum	*0.55 ± 0.08^c^	*0.81 ± 0.02^bc^	*0.93 ± 0.08^b^	*1.56 ± 0.12^a^
Total Ig (mg ml^–1^)	Mucus	0.25 ± 0.02^b^	0.27 ± 0.01^b^	0.31 ± 0.01^b^	0.41 ± 0.02^a^
Lysozyme (U mg^–1^ protein)	Serum	*19.76 ± 0.90^ab^	*18.73 ± 0.31^b^	*20.50 ± 0.32^ab^	*21.20 ± 0.11^a^
Lysozyme (U mg^–1^ protein)	Mucus	8.70 ± 0.30^b^	8.70 ± 0.20^b^	9.16 ± 0.08^b^	10.16 ± 0.20^a^
Sod (U mg protein^–1^)	Homogenized-fish	2270.46 ± 11.55^d^	2355.33 ± 14.43^c^	2476.66 ± 17.63^b^	2605.33 ± 16.38^a^
Cat (U mg protein^–1^)	Homogenized-fish	0.20 ± 0.01	0.27 ± 0.37	0.26 ± 0.01	0.30 ± 0.02^a^

Data presented as the mean ± S.E.

Different small letters denote significant differences among treatments (*P* < 0.05).

Significantly different concentrations between mucus and serum are denoted by asterisks (*P* < 0.05).

In Table 3, the effects of dietary supplementation on the antioxidant defenses of zebrafish are shown. SOD activity was significantly increased in fish fed with different doses of nutmeg, with fish fed with a T3-nutmeg diet showing the highest SOD activity (*P* < 0.05). Cat activity was not affected by the dietary nutmeg supplementation.

### 3.3. Growth, immune, and antioxidant mRNA expression

**FIGURE 1:**
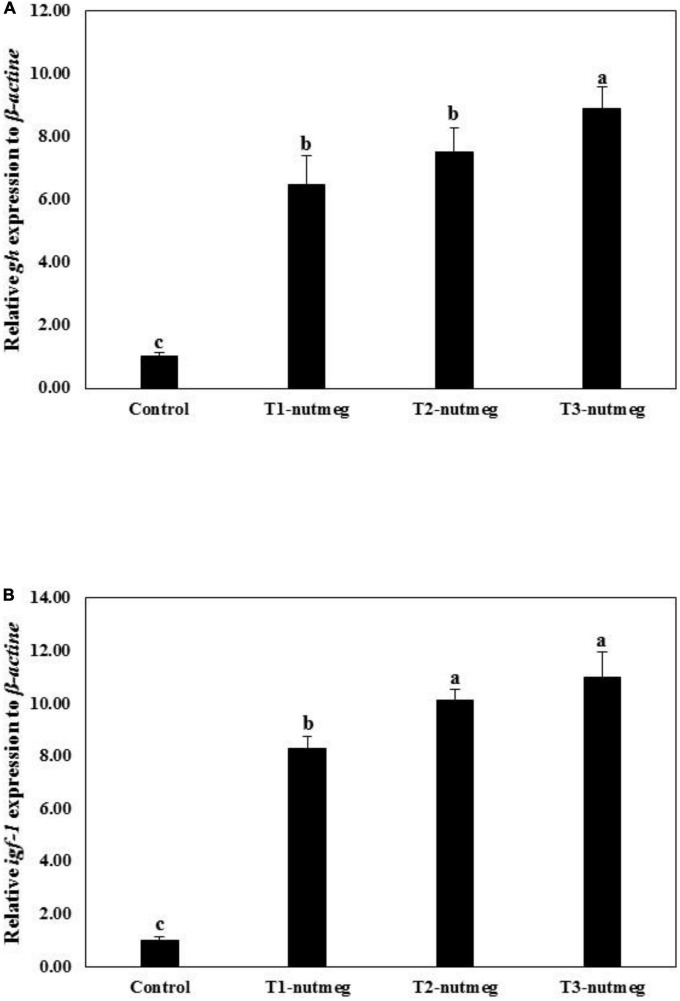
*Growth hormone* (*gh*) **(A)** and *insulin growth factor-1* (*igf-1*) **(B)** gene expression of zebrafish fed the experimental diets (mean ± S.D.). Bars assigned with different small letters denote significant differences among treatments (*P* < 0.05).

**FIGURE 2:**
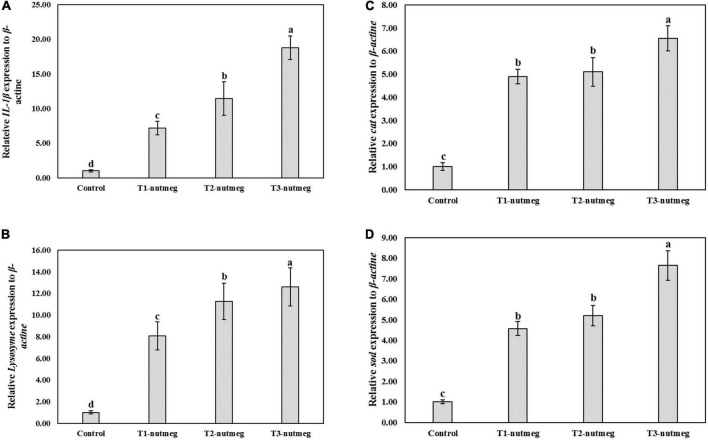
*Intelerkin-1*β (*IL-1*β) **(A)**, *lysozyme* **(B)**, *catalase* (*cat*) **(C)**, and *superoxide* dismutase (*sod*) **(D)** gene expression of zebrafish fed the experimental diets (mean ± S.D.). Bars assigned with different small letters denote significant differences among treatments (*P* < 0.05).

Figure 1 presents the expression of brain *gh* and hepatic *igf*-1 of zebrafish fed with nutmeg-supplemented diets. Nutmeg-supplemented diets considerably upregulated the expression of *growth-related* genes in the brain and liver (*P* < 0.05). The T3-nutmeg diet with the highest expression value and a control diet with the lowest expression value were observed, respectively. The intestinal mRNA expression of *IL-1*β and *lysozyme* of zebrafish fed with nutmeg-supplemented diets are presented in Figure 2. Both *IL-1*β and *lysozyme* expressions were significantly increased in intestine tissue of zebrafish as doses of nutmeg were increased (*P* < 0.05). In terms of antioxidant genes, both hepatic *sod* and *cat* were significantly upregulated in fish fed with nutmeg dietary, and the highest value was observed for T3-nutmeg in comparison with the control (*P* < 0.05).

### 3.4. Coldwater stress tolerance

**FIGURE 3:**
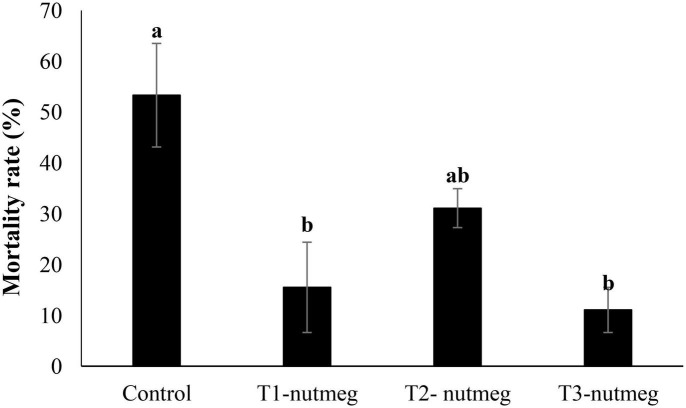
The mortality rate of zebrafish fed the experimental diets and exposed to the cold-water challenge (13 ± 2°C) (mean ± S.D.). Bars assigned with different small letters denote significant differences among treatments (*P* < 0.05).

A diet supplemented with nutmeg at 2 and 3% significantly decreased zebrafish mortality after cold-temperature shock (Figure 3). The death rate of fish was the highest, among fish derived from the control group.

## 4. Discussion

Plants are being studied as immunomodulators, especially for improving fish performance, immune systems, and disease resistance, thus it has long been recognized that medicinal herbs and spices are among the most important targets (34–36). Medicinal herbs, as eco-friendly substances, provide a variety of benefits, including growth and resistance promoters against environmental shocks, thermal and pollution stress, and parasites (1, 6, 12, 27–30). In this sense, our current study was conducted to evaluate fish growth performance after feeding powdered nutmeg seed, following our previous studies on zebrafish fed with plants, namely ferula (*Ferula assafoetida*) (37), myrtle (*Myrtus communis*) (38), and coriander (*Coriandrum sativum*) (30). In addition to examining the antioxidant activity and immune response of zebrafish, we examined some related gene expressions of fish fed with nutmeg dietary supplementation. Our results indicated an improvement in zebrafish non-specific immunity and antioxidant capacity. The high activity of lysozyme and total Ig besides upregulation of *IL-1*β, *lysozyme*, *sod*, and *cat* mRNA expressions showed a positive impression of nutmeg-supplemented diets on the immune function of zebrafish.

In the current study, a nutmeg-supplemented diet did not significantly improve the fish growth performance; however, the transcription of growth-related genes in the brain and liver of zebrafish was considerably upregulated using different doses. The same results from our previous publication, dietary *Dragonhead kotschyi* supplementation on zebrafish in the same condition (39), confirmed that plant-based diets might promote immunity rather than growth in this species. Stoev et al. (24) showed that nutmeg negatively affected the growth performance and survival of rainbow trout (*Oncorhynchus mykiss*). Moreover, Zhelyazkov et al. (40) reported insignificant live weight of common carp (*Cyprinus carpio*) between fish fed with 1% nutmeg and control. On the contrary, Rashidian et al. (25) reported the highest conclusion of growth factor in common carp-fed nutmeg supplementary diets. Growth hormone (GH) is a potent growth promoter either itself or through stimulating the liver to synthesize and release the insulin-like growth factor (IGF-I) which impacts protein metabolism (41). Due to the fact that fish were previously fed for almost 8 weeks, it might be the duration of feeding that could also play an important role in performance. Moreover, there is different feedback based on species, as 12 week feeding trial of nutmeg extract in rats might be an actor to increase muscle mass, thus nutmeg treatment induced the muscle IGF-I expression (42). GH and IGF are critical factors that control the teleost’s growth (43) and have a positive relationship with growth factors when it comes to using medicine herbs (44). In our study, although there is an upregulation of GH and IGF expressions, there is no significant difference in the weight gain of zebrafish that may be nutmeg diet-additive for health-promoting purposes in fish.

The results of the present study also revealed the innate immune improvement of zebrafish when fed with nutmeg-supplemented diets. In response to nutmeg inclusion in the diet, innate-immune parameters including total protein, total Ig, and lysozyme in both mucus and serum were significantly elevated. Moreover, the transcripts of immune-related genes, that is, *IL-1*β and *lysozyme*, were remarkably upregulated in fish fed with different doses of nutmeg powder, similar to other plant- and algae-supplementary diets application to zebrafish, *D. kotaschyi* (39) and *Ulva intestinalis* (45). Lysozyme acts as the main antibacterial enzyme by catalyzing the hydrolysis of 1,4-beta-linkages in the peptidoglycan of gram-positive bacteria, or indirectly against gram-negative bacteria (46). An *in vitro* study has found nutmeg extract to be an inflammatory activator (20). In addition to its anti-inflammatory functions, nutmeg also contains analgesic properties, and compounds that possess analgesic and anti-inflammatory capabilities may also possess antibacterial qualities (47). The *in vitro* study on the bioactive compound of nutmeg revealed that a high portion belongs to sabinene (38%). Interestingly, it has been shown that sabinene has strong antimicrobial and immune-enhancing effects (48, 49). The beneficial effects of nutmeg on immune parameters can be attributed to the presence of such a bioactive compound.

Increasing SOD activity along with the upregulation of *sod* and *cat* genes might indicate that nutmeg additives into the diets of zebrafish could improve its antioxidant capacity. Nutmeg’s positive effects on the limitation or inhibition of oxidation possibly are due to the tribonoid, phenolic, and flavonoid compositions of nutmeg additive. As antioxidants, these compounds eliminate oxygen-free shirts, metals, and radicals (50). Based on the study of the bioactive compound of nutmeg, sabinene was the most frequent compound. It has been reported that this compound has strong antioxidant activity (51). The highest effectiveness of nutmeg through immune and antioxidant activities was observed at 2% and 3% herb-additive (25). Regarding our results, *Cat* activity in zebrafish was not significantly different between fish fed with doses of nutmeg. The presence of plant-nutritional properties on fish mucus is also reported in publications related to innate immunity and antioxidant activity (52–55). The mortality rate of fish fed with 1% and 3% nutmeg decreased significantly after cold-shock stress of 4 h. Previously, heat stress alleviated after nutmeg administration confirmed the antioxidant contribution of this plant (23). In zebrafish fed different medicinal plants, the highest antioxidant activity was observed in cases of cinnamon and clove extracts, had the highest antioxidant activity, *i.e.*, the most effective inhibitors of copper-mediated LDL oxidation and macrophage phagocytosis (56). In contrast, when fish were treated with clove extract, their growth performance was the least improved. The antioxidant activity might indicate fish resistance capacity, especially in fish exposed to infectious, destructive agents and stressors (57). In this sense, the higher SOD activity and also the higher *sod* and *cat* expressions that are observed in fish fed with nutmeg-supplemented diets led to lower mortality after the stress tolerance challenges. A diet with 3% nutmeg powder also demonstrated immune-promoting potential, making it the suggestive diet. In conclusion, our results revealed that powdered *M. fragrans* seeds supplementation significantly enhanced immune and antioxidant functions in zebrafish diets. Nutmeg inclusion diets upregulated *IL-1*β and *lysozyme*, immune modulator enzymes, and fish resistance to water-temperature stress. However, further studies are required to know the exact mechanism of how nutmeg regulated the non-specific immune response of teleosts based on the specific physiological reactions of species.

## Data availability statement

The original contributions presented in this study are included in this article/supplementary material, further inquiries can be directed to the corresponding author.

## Ethics statement

The animal study was reviewed and approved by the GUASNR.

## Author contributions

FV, ZR, RS, MR, and SH contributed to the methodology, laboratory, and data analysis. ZR, RS, MR, MH, IG, AA, and SH wrote and developed the manuscript. RS supervised the project. All authors contributed to the article and approved the submitted version.
