# Corrigendum: Effects of dietary nutmeg (*Myristica fragrans*) seed meals on growth, non-specific immune indices, antioxidant status, gene expression analysis, and cold stress tolerance in zebrafish (*Danio rerio*)

**DOI:** 10.3389/fnut.2023.1210933

**Published:** 2023-05-22

**Authors:** Farzaneh Vakili, Zahra Roosta, Roghieh Safari, Mojtaba Raeisi, Md. Sakhawat Hossain, Inês Guerreiro, Arash Akbarzadeh, Seyed Hossein Hoseinifar

**Affiliations:** ^1^Department of Fisheries, Sari Agricultural Sciences and Natural Resources University, Sari, Iran; ^2^Fisheries Department, Faculty of Natural Resources, University of Guilan, Someh Sara, Gilan, Iran; ^3^Department of Fisheries, Faculty of Fisheries and Environmental Sciences, Gorgan University of Agricultural Sciences and Natural Resources, Gorgan, Iran; ^4^Food, Drug and Natural Products Health Research Center, Golestan University of Medical Sciences, Gorgan, Iran; ^5^Hagerman Fish Culture Experiment Station, University of Idaho, Hagerman, ID, United States; ^6^CIIMAR - Interdisciplinary Centre of Marine and Environmental Research, Terminal de Cruzeiros do Porto de Leixões, University of Porto, Matosinhos, Portugal; ^7^Department of Fisheries, Faculty of Marine Science and Technology, University of Hormozgan, Bandarabbas, Iran

**Keywords:** nutmeg, growth and immune related genes, oxidative stress, coldwater stress challenge, medicinal herb

In the published article, an author name was incorrectly written as “Arash Akbazadeh.”

The correct spelling is “Arash Akbarzadeh.”

The authors apologize for this error and state that this does not change the scientific conclusions of the article in any way. The original article has been updated.

